# Combined positron emission tomography and contrast enhanced CT (PET/CeCT) is a feasible single investigation in the staging of oesophagogastric cancers: single-centre pilot study experience during the COVID-19 pandemic

**DOI:** 10.1308/rcsann.2023.0070

**Published:** 2023-11-20

**Authors:** M Jones, S Higgs, S Dwerryhouse, V Markos, K Mason, C Green, A Nawwar, J Searle, I Lyburn

**Affiliations:** ^1^ Gloucestershire Hospitals NHS Foundation Trust, UK; ^2^ Cobalt Medical Charity, UK

**Keywords:** Oesophageal neoplasms, Stomach neoplasms, Positron emission tomography, Tomography, X-ray computed, Neoplasm staging

## Abstract

**Introduction:**

Staging of oesophagogastric (OG) cancers usually involves endoscopy (OGD), and separate visits for contrast enhanced computed tomography (CeCT) and positron emission tomography (PET/CT). At the height of the COVID-19 pandemic, some of our patients underwent single-visit combined staging with PET/CeCT. We compare this novel pathway with standard separate imaging in time to completion of staging, to start of treatment, and cost.

**Methods:**

We identified all patients discussed at our OG multidisciplinary team (MDT) meeting in 2020. Clinical records revealed dates of investigations and treatments. Data were tabulated in Excel, with statistical analysis in SPSS. All patients followed the same MDT process and image reviewing criteria. Costs were compared using prices supplied by finance departments.

**Results:**

A total of 211 new patients were discussed at our MDT in 2020. Of these, 48 patients had combined PET/CeCT staging, and 68 had separate scans. Median time (interquartile range) in days from OGD to final imaging was 9 (6–23) for the combined group versus 21 (16–28) for the separate group (*p*≤0.001). Median time (days) from OGD to treatment start was 37 (29–52) for combined versus 55 (40–71) for separate (*p*≤0.001). No combined scans were of insufficient diagnostic quality for the MDT. PET/CeCT had a potential cost saving of £113 per patient.

**Conclusions:**

PET/CeCT allows accurate radiological staging of OG cancers with a single scan. Patients completed staging and started treatment faster, with a potential saving of £10,509 in one year. PET/CeCT has become standard staging at our trust, and we aim to incorporate radiotherapy planning images too.

## Introduction

Oesophagogastric (OG) cancers are associated with significant morbidity and mortality. The National OG Cancer Audit 2022 (NOGCA) reported 19,174 patients diagnosed with OG cancers between 1 April 2019 and 31 March 2021 in England and Wales. Patients are commonly referred through the Two-Week-Wait suspected cancer pathway, but some present acutely to hospital (often with symptoms related to advanced disease).^1,2^

Once a diagnosis of OG cancer has been made, accurate staging is essential to allow the formulation of appropriate treatment plans, and to advise on prognosis. This process involves multiple hospital visits that have financial, emotional and time-related implications for patients, family members and caregivers.

The COVID-19 pandemic had a profound effect on the provision of cancer care. Attendances to hospital involved potential exposure to COVID, with cancer patients being especially vulnerable due to their relative immunosuppression.^3–5^ Therefore, reduced exposure to the healthcare environment could only be beneficial for these patients and the overall healthcare system.

Cross-sectional imaging is vital in assessing for metastatic disease in OG cancers. In addition to high-resolution intravenous contrast-enhanced computed tomography (CT) (CeCT), ‘standard’ positron emission tomography (PET/CT) (F-18 fludeoxyglucose F18 (FDG) PET with noncontrast enhanced low-dose CT) is employed because around 15% of patients present with occult metastatic disease not seen on CT alone.^6^ Use of PET/CT is recommended for staging of oesophageal and OG junctional cancers in the UK (in those patients who may be suitable for radical treatment or to help direct palliative therapy) or those with gastric cancer in whom metastatic disease may be suspected.^7^

Endoscopic ultrasound can play a role in assessing depth of invasion of oesophageal tumours and local lymph node involvement but in many centres can be a limited resource. In addition, diagnostic laparoscopy may be employed to assess the peritoneal cavity for metastatic disease and assess resectability of a primary tumour. NICE guidance recommends that both endoscopic ultrasonography (EUS) and staging laparoscopy should be offered to patients only when it would help with ongoing management.^7^

The pandemic prompted drastic modifications to the investigation and treatment of OG cancers at our regional subspecialist centre. In our unit, this included wholesale changes to the staging pathway. During the first lockdown, we made the decision to limit endoscopic ultrasound and staging laparoscopy only to those patients in whom it would make a fundamental difference to their treatment pathway, to balance the risk of aerosol-generating procedures to patients and staff. NOGCA demonstrated that this type of change was widespread in England and Wales, with only 18.6% of patients having EUS in 2020/2021 compared with 28% in 2019/2020.^1^

We also decided to trial a novel single-staging PET scan with diagnostic high-resolution intravenous CeCT (PET/CeCT) for OG cancers, which could combine staging image acquisition into one sitting. There is no published literature on the use of combined PET/CeCT in the staging of OG cancers, but its use is reported for other malignancies. Cross-modality image fusion of PET/CT and CeCT has been described as effective in the diagnosis and staging of pancreatic cancer, compensating for the perceived shortcomings of PET/CT and CeCT alone.^8^ It was found to be highly sensitive (90%) in lung cancer, providing a feasible technique for staging and restaging.^9^ It has also been shown to have high sensitivity and specificity in surveillance of patients post colorectal cancer surgery who have asymptomatic elevation in carcinoembryonic antigen levels.^10^ In medullary thyroid cancer, high sensitivity has been reported, particularly in detecting lymph node metastases when compared with traditional ultrasonography.^11^

By adopting this innovative combined PET/CeCT pathway, we aimed to provide accurate and prompt staging while reducing the number of hospital visits for our patients. We assessed whether the new PET/CeCT pathway had any influence on time to completion of staging, time to start of treatment and whether there were potential financial savings for the NHS when compared with the standard staging pathways of separate imaging.

## Methods

This was a retrospective single-centre study. All patients had signed consent for use of their anonymised data in audit and publication. We identified those who had been discussed in the OG multidisciplinary team (MDT) meeting between 1 January and 31 December 2020. MDT records, clinical letters, endoscopy reports and radiology systems were reviewed. Data were tabulated in Microsoft Excel, and SPSS (IBM) was used to perform two Mann–Whitney *U* tests to compare the combined imaging group with the separate imaging group (time from OGD to final imaging, and time from OGD to start of treatment).

### Combined group (PET/CeCT)

Scans took place between 31 March and 17 November 2020 at a fixed-site PET/CT scanner provided by Cobalt Medical Charity (CMC), where PET/CT has been undertaken since 2006. There was no change in the physical pathway of the patient, and only the addition of intravenous (IV) contrast and modification of scan parameters was required to undertake PET/CeCT. Images were interpreted by consultant radiologists as per usual practice.

All patients having combined PET/CeCT received their diagnosis in the host OG Cancer Centre. Patients referred from other regional trusts who were discussed at the MDT were not eligible for combined imaging due to funding stream availability at the host trust only. Instead, they went through the standard staging imaging pathway.

Conventional PET/CT scans were paid for by NHS England as part of the national contract in the standard patient pathway. We were able to substitute the low-dose non-contrast CT with an intravenous CeCT at the PET/CT appointment slot without adding extra time to the scan, avoiding a separate three-part CT scan.

### Separate group (PET/CT+CT)

Patients undergoing PET/CT + separate CeCT following a diagnostic OGD were included in this group, which was considered the standard pathway for workup of newly diagnosed OG cancers and is in line with UK guidance.

### CT-only group

This group comprised patients who had a CeCT only (after diagnostic OGD). This group probably self-selects to CT only, as the method of presentation is frequently through the emergency pathway, and often no further staging investigations are necessary in patients with poor performance status and metastatic disease on CT.

### Excluded patients

For the purposes of this study, patients who did not start their pathway with a diagnostic OGD were excluded. Non-epithelial tumours were also excluded (this accounted for one patient with a gastrointestinal stromal tumour), along with patients for whom imaging records could not be obtained from other trusts or MDT documentation was not completed.

### Pathways

We used the diagnostic OGD as the starting point of the investigation pathway, as this accounts for most patients with OG cancers. We assessed the difference in median (interquartile range) time to completion of staging by comparing the number of days from visual confirmation/suspicion of malignancy at diagnostic OGD to final imaging. The date at which treatment (either curative or palliative) commenced was used to compare the median time to start of treatment between the groups.

We also recorded the numbers of patients revealed to have metastatic disease on staging imaging, and how many patients went down a treatment pathway of curative intent, or palliative. To assess accuracy of staging imaging, we assessed the proportion of patients per group who had a pathological T or N stage higher than that described on the staging imaging.

### Imaging

The technical aspects of PET/CeCT imaging were advised on by consultant radiologists in the OG cancer MDT. The CT acquisition parameters were modified, with slice thickness altered from 3mm to 1mm with acquisition during gentle breathing commencing 70 seconds after the start of IV contrast administration. We assessed the quality of imaging objectively, by reviewing MDT outcomes and looking to see whether any patients in the PET/CeCT group required repeat scans due to suboptimal CT component diagnostic quality. All imaging went through the same MDT reporting process.

### Finances

We liaised with our trust Finance Department for expenditures. At the host OG centre, PET/CT scans are paid for by NHS England as part of a national contract and the exact cost could not be shared with us. However, national pricing for a ‘standard’ PET/CT was made available, which is around £750 per scan including the cost of the isotope. According to the National Institute for Health Research ‘Interactive Costing Tool’ Investigation and Intervention Tariff 2020/2021, the cost of a standalone contrast enhanced CT chest, abdomen and pelvis at national tariff plus market forces factor is £113 irrespective of whether IV contrast material is used.^12^ The CeCT component of the PET/CeCT was provided free of charge by CMC as part of the pilot study.

## Results

A total of 211 patients with new diagnoses in 2020 were discussed in our MDT over the course of the year. Of these, 160 patients met the study inclusion criteria; 48 patients (30%) had combined PET/CeCT; 68 patients (43%) went through the standard separate imaging pathway of PET/CT and CeCT; and 45 patients (28%) had CT only.

Median age was 70 years (63–77) in the separate CT+PET/CT group, and 72 years (61–77) in the combined imaging group. Median age was 81 (74–87) for the CT alone group.

### Distribution of tumours

**Figure 1:**
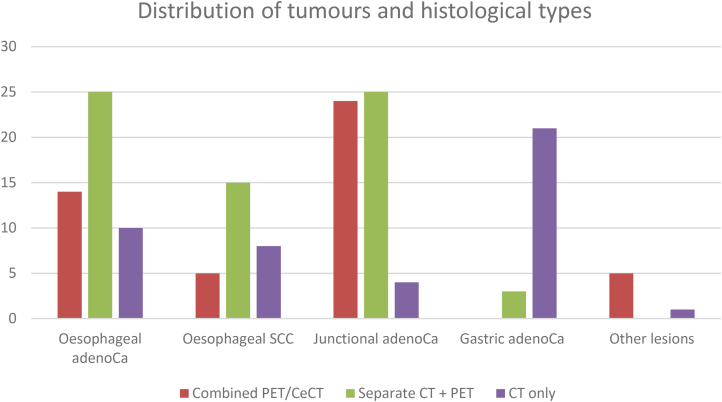
Distribution of tumours and histological types (adenoCA = adenocarcinoma; CeCT = contrast-enhanced CT; CT = computed tomography; PET = positron emission tomography; SCC = squamous cell carcinoma).

A summary of the distribution of tumours is displayed below in Table 1 and Figure 1.

**Table 1:** Distribution of tumours and histological types

Pathway	Oes ACA	Oes SCC	GOJ ACA	Stomach ACA	Other lesions
Combined PET/CeCT [*n*=48]	Prox: 0 Mid: 1 Dist: 13	Prox: 0 Mid: 3 Dist: 2	Type 1: 10 Type 2: 8 Type 3: 6		Oesoph: 4 benign Gastric: 1 benign
Separate CT+PET [*n*=68]	Prox: 0 Mid: 2 Dist: 23	Prox: 2 Mid: 7 Dist: 6	Type 1: 10 Type 2: 5 Type 3: 8 Unspec: 2	Prox: 0 Mid: 2 Dist: 1	
CT only [*n*=44]	Prox: 0 Mid: 2 Dist: 8	Prox: 2 Mid: 3 Dist: 3	Type 1: 2 Type 2: 1 Type 3: 1	Prox: 7 Mid: 5 Dist: 9	Lower oesoph small cell: 1

CeCT = contrast-enhanced CT; CT = computed tomography; Dist = distal; GOJ ACA = gastro-oesophogeal junction ACA; Mid = ; Oes ACA = oesophageal adenocarcinoma; Oes SCC = oesophageal squamous cell carcinoma; Oesoph, oesophageal; stomach ACA = stomach adenocarcinoma; PET = positron emission tomography; Prox = proximal

### Time to completion of staging

In the separate group, the median number of days (interquartile range) between diagnostic OGD and CT was 9 (5–14). Median time from CT to PET was 15 (11–20) days.

Overall, the median time from diagnostic OGD to completion of staging was 21 (16–28) days for the separate group, compared with 9 (6–23) for the combined group (*p*<0.001)

For the CT-only group, median time from OGD to CT was 8 (3–16) days.

### Time to start of treatment

**Figure 2:**
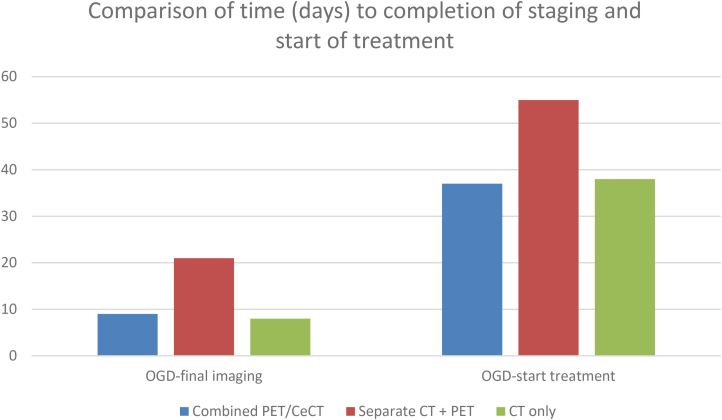
Comparison of median times (interquartile range) in days from diagnostic OGD to imaging and treatment start dates. (OGD = oesophagogastric endoscopy)

The median time from diagnostic OGD to start of treatment (this could be radical or palliative) was 37 days (29–52) in the combined group versus 55 days (40–71) for the separate group. For the Mann–Whitney *U* Test, five patients from the combined group were excluded from the calculation as they did not start any treatment due to no malignancy being found (*p*<0.001). Median time from OGD to start of treatment was 38 days (25–51) in the CT only group Table 2 and Figure 2.

**Table 2:** Comparison of median times (interquartile range) in days from diagnostic OGD to imaging and treatment start dates

	OGD-CT	CT-PET	OGD-final imaging	OGD-start treatment
Combined PET/CeCT [*n*=48]	–	–	9 (6–23) [*n*=48]	37 (29–52) [*n*=43]
Separate CT+PET [*n*=68]	9 (5–14)	15 (10–20)	21 (16–28)	55 (40–71)
CT only [*n*=44]	8 (3–16)	–	8 (3–16)	38 (25–51)

CeCT = contrast-enhanced CT; CT = computed tomography; OGD = oesophagogastric endoscopy; PET = positron emission tomography

### Quality of PET/CeCT images

The MDT did not find any combined PET/CeCT to be suboptimal or substandard for diagnosis, and none needed to be repeated.

### Metastatic disease and treatment outcomes

In the combined group, 11 patients (23%) were staged as having M1 disease on the TNM system. This compares with 15 (22%) in the separate group and 19 (43%) in the CT only group. In the complete cohort of 160 patients, this would put the proportion of patients with metastatic disease at presentation at 28% (lower than the 43% rate reported by NOGCA in England and Wales).

In the combined group, 14 patients (41%) had treatment with curative intent, 15 (44%) had palliative and 5 (15%) required no treatment as they were shown not to have malignancy.

For the separate group, 38 patients (57%) had treatment with curative intent and 29 (43%) palliative. For the group having CT alone, 6 (13%) patients had treatment with curative intent and 39 (87%) palliative (Table 3).

**Table 3:** Proportions of patients with metastatic (M1) disease on imaging, and treatment with radical or palliative intent

		Treatment based on imaging		Patients having laparoscopy	Upstaging based on laparoscopy
	Metastatic disease on imaging	Radical	Palliative		
Combined PET/CeCT [*n*=48]	23%	50%	50%	19%	4% (*n*=1)
Separate CT+PET [*n*=68]	22%	56%	44%	21%	0%
CT only [*n*=44]	40%	7%	93%	9%	0%

CeCT = contrast-enhanced CT; CT = computed tomography; PET = positron emission tomography

### Radiological versus pathological staging accuracy

In the combined PET/CeCT group, 38% of patients went on to have surgery with curative intent, with 100% of those having neo-adjuvant therapy. This compared with 43% in the separate group, with 90% having neo-adjuvant treatment. In the CT-only group, 7% of patients had surgery with curative intent, with 0% receiving neo-adjuvant treatment.

In the combined group, only one patient (6%) had a histological T stage higher than demonstrated radiologically on the staging scan. This patient had an Ivor-Lewis oesophagectomy for a type 1 junctional adenocarcinoma. Three patients (18%) had histological N stages higher than reported on the staging PET/CeCT. They had Ivor-Lewis oesophagectomies for type 1 or type 2 junctional adenocarcinoma.

In the separate group, five patients (18%) had a histological T stage higher than predicted on staging imaging. Their tumours included one lower oesophageal adenocarcinoma, three junctional adenocarcinomas and one midgastric adenocarcinoma. The lower oesophageal and junctional adenocarcinomas had all received neoadjuvant chemoradiotherapy, and the gastric cancer had undergone neoadjuvant chemotherapy. Five patients (18%) had a histological N stage higher than predicted on imaging.

There was one patient in the separate group who had an open/close operation. They had a type 3 junctional adenocarcinoma and had undergone neoadjuvant chemotherapy. At surgery they were found to have a T4b tumour involving the aorta.

In the CT only group, one patient (33%) with a distal gastric adenocarcinoma had a histological T and N stage higher than that demonstrated on imaging. This patient had not received neoadjuvant treatment (Table 4).

**Table 4:** Comparison of radiological vs pathological T&N stages for patients having radical surgery

	Combined PET/CeCT (*n*=18)	Separate CT+PET (*n*=28)	CT only (*n*=3)
Neoadjuvant treatment	100%	90%	0%
Pathological T stage>than radiological	6%	18%	33%
Pathological N stage>than radiological	18%	18%	33%
Open/close rate	0%	4%	0%

CeCT = contrast-enhanced CT; CT = computed tomography; PET = positron emission tomography

### Costs

By completing the PET and CeCT component in one sitting (within the same timeframe and requiring only the addition of IV contrast), the need for a separate three-part contrast enhanced CT scan at £113 was avoided. Given that the National Institute for Health and Care Research (NIHR) interactive costing tool 2020 states no price difference for adding IV contrast to a CT chest/abdomen/pelvis, it must be assumed that adding IV contrast for the combined PET/CeCT incurs no difference in cost.

For an estimate of cost savings, we added the 68 patients in the separate group to the 48 in the combined group, giving a total of 116 patients who were planned to have PET imaging as per the MDT. We then felt it was pragmatic to assume that 20% of these patients would not have gone on to complete a PET scan (due to having significant metastatic disease visible on CT alone or being unfit/choosing not to have active treatment); this would leave 93 patients remaining. Scaling up £113 (the cost avoided for a standalone contrast CT scan) for each of these 93 patients would provide a potential financial saving of £10,509 over the course of a year for our NHS trust.

**Figure 3:**
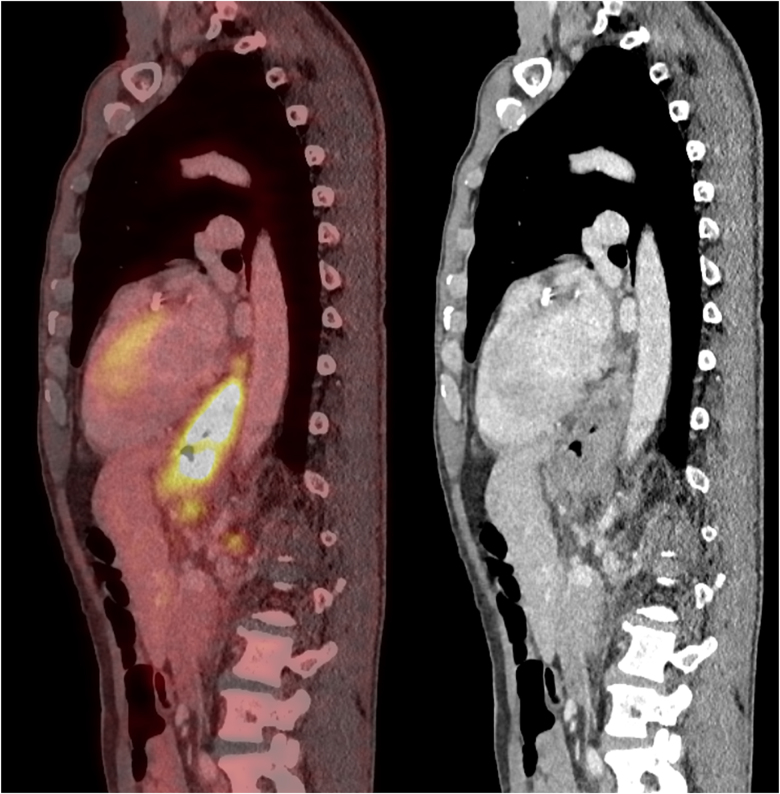
Siewert Type 2 primary lesion on sagittal PET/CeCT (CeCT = contrast-enhanced CT; CT = computed tomography; PET = positron emission tomography)

**Figure 4:**
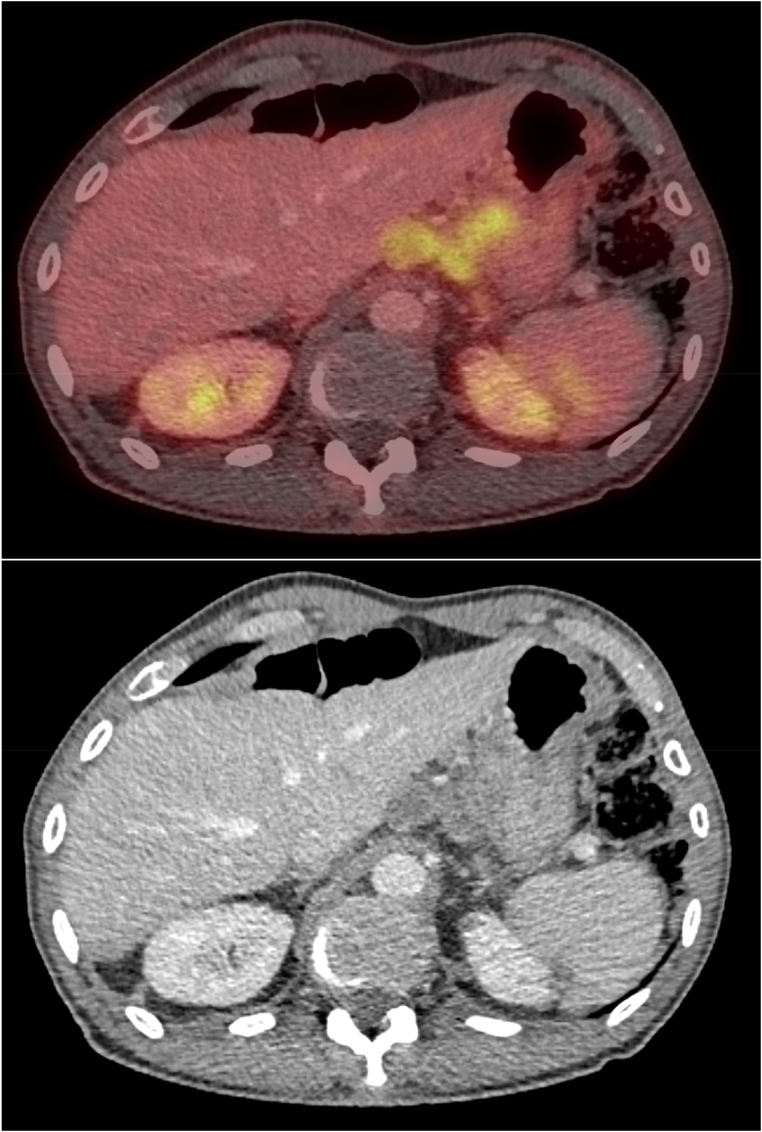
Siewert Type 2 left gastric nodes on PET/CeCT (CeCT = contrast-enhanced CT; CT = computed tomography; PET = positron emission tomography)

**Figure 5:**
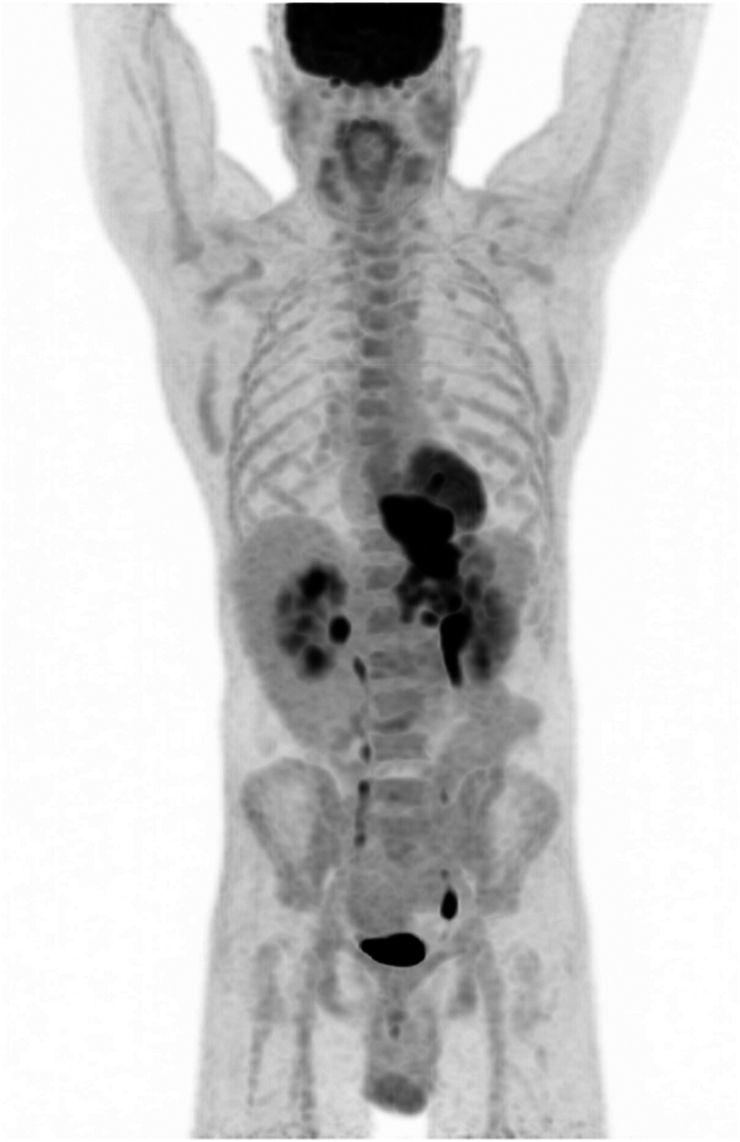
Siewert Type 2 oesophageal cancer MIP PET (MIP = maximum intensity projection; PET = positron emission tomography)

Reduced financial implications for patients, family members and carers must also be considered, given less travel time and associated costs, along with less absence from work Figures 3–5.

## Discussion and conclusions

The experience we gained with combined PET/CeCT during the COVID pandemic has fostered what we feel to be a positive change in our staging pathway for OG cancers. We found that combined imaging provided a more streamlined and convenient staging process. It allowed patients to complete their diagnostic imaging faster with a single visit, and to start their treatment more rapidly, with statistical significance demonstrated.

We feel combined imaging is preferable for patients and their caregivers, and more cost efficient for our hospital trust (and the wider health service) when compared with the standard separate pathway, with significant potential savings when scaled up to include all those who would go through the standard separate pathway. There are also perceived time benefits to staff and services given that the investigation requires a single referral request, and radiologists have only to access one group of images when reporting PET/CeCT.

We have continued other modifications first employed during the pandemic, namely around the use of EUS. We now use it very selectively, where it will make a significant difference to management, for example early T1b versus T2 oesophageal cancer, and occasionally in patients where there is debate about location of junctional tumours.

There is the question of whether PET/CeCT is necessary for those who would have metastatic disease visible on CT alone. For this group of patients, using PET may be seen as unwarranted. However, the opinion of our MDT is that for patients not presenting as emergencies, and even for patients with obvious metastatic disease on CT, combined imaging has proved very useful when planning palliative oncology treatment. It did not prolong the staging process and we feel it is justified as a standard investigation in the combined pathway.

It is difficult to make a direct comparison of combined PET/CeCT with existing data, as there are no published studies on its use in upper gastrointestinal (GI) malignancy and we do not know of any other UK centres that have yet implemented it. We feel this study is therefore beneficial to highlight its potential benefits.

### Limitations

This is a retrospective analysis of a small group of patients on a novel upper GI cancer staging pilot study. Further research on the efficiency and diagnostic value of combined PET/CeCT in OG cancers is required, and a randomised trial or prospective longitudinal study may bring more evidence in future.

Other changes to the staging pathway during this period, such as the modified use of EUS, will have influenced the overall time to treatment compared with before the pandemic, but this does not affect this study, where all groups were affected equally.

The contrast CT component of the combined PET/CeCT scans in this project was provided free of charge by a medical charity as part of the pilot study. However, no non-clinical employee of the charity had any role in reviewing imaging, MDT discussion or decision-making for any patient.

Combined PET/CeCT scans were available only to patients in the host trust, solely due to the funding stream limitations for this pilot study, and a risk of bias must therefore be noted in the turnaround time for the PET/CeCT group. Despite this, all regional referrals from outside the host trust went through the standard staging pathway and received the same standards of care in the MDT.

## Conclusions

In this study, combined PET/CeCT seems to allow rapid and accurate radiological staging of OG cancers in a single patient visit. We noted a reduced time to treatment, and cost saving of £113 per scan when compared with separate staging imaging. Outside of the pandemic scenario, combined PET/CeCT is now being used as standard in our MDT pathway for OG cancers and, given its benefits in planning both radical and palliative treatment, we feel its use is justified even for those who would have metastatic disease demonstrated on CT alone. We are considering acquiring radiotherapy planning CT images in the same episode (by using a flatbed couch) in future to further shrink the patient pathway. Further studies are required to assess the efficiency and diagnostic value of combined PET/CECT in upper GI cancer diagnosis.

## Ethical compliance

All patients had signed consent for use of their anonymised data in audit and publication.

## Data access statement

Raw anonymised data have been supplied to reviewers and can be made available on request to readers.

## Conflict of interest declaration

The authors have no conflicts of interest to declare. Four of the authors have NHS sessions seconded to Cobalt Medical Charity who provides the PET/CT scanner, as per standard practice for 16 years.

## References

[1] Healthcare Quality Improvement Partnership (HQIP). *National oesophago-gastric cancer audit 2022*. https://www.hqip.org.uk/a-z-of-nca/national-oesophago-gastric-cancer-audit/ (cited August 2023).

[2] Sharpe D, Williams RN, Ubhi SS *et al.* The ‘two-week wait’ referral pathway allows prompt treatment but does not improve outcome for patients with oesophago-gastric cancer. *Eur J Surg Oncol* 2010; **36**: 977–981. 20702059 10.1016/j.ejso.2010.07.002

[3] Liu C, Zhao Y, Okwan-Duodu D *et al.* COVID-19 in cancer patients: risk, clinical features, and management. *Cancer Biol Med* 2020; **17**: 519–527. 32944387 10.20892/j.issn.2095-3941.2020.0289 PMC7476081

[4] Kamboj M, Sepkowitz K. Nosocomial infections in patients with cancer. *Lancet Oncol* 2009; **10**: 589–597. 19482247 10.1016/S1470-2045(09)70069-5

[5] Sidaway P. COVID-19 and cancer: what we know so far. *Nat Rev Clin Oncol* 2020; **17**: 336. 10.1038/s41571-020-0366-2 PMC7136993 32265531

[6] Cummings D, Wong J, Palm R *et al.* Epidemiology, diagnosis, staging and multimodal therapy of esophageal and gastric tumors. *Cancers (Basel)* 2021; **13**: 582. 33540736 10.3390/cancers13030582 PMC7867245

[7] National Institute for Health and Care Excellence (NICE). *Oesophago-gastric cancer: assessment and management in adults*. NICE Guideline NG 83 2018. https://www.nice.org.uk/guidance/ng83 (cited September 2023).

[8] Zhang J, Zhuo CJ, Jia NY *et al.* Cross-modality PET/CT and contrast-enhanced CT imaging for pancreatic cancer. *World J Gastroenterol* 2015; **21**: 2988–2996. 25780297 10.3748/wjg.v21.i10.2988 PMC4356919

[9] Nanni C, Rossetti V, Zompatori M *et al.* Performance of FDG PET/CeCT in the evaluation of patients with lung cancer. *Biomed Pharmacother* 2014; **68**: 219–223. 24486108 10.1016/j.biopha.2013.11.002

[10] Vallam KC, Guruchannabasavaiah B, Agrawal A *et al.* Carcinoembryonic antigen directed PET-CECT scanning for postoperative surveillance of colorectal cancer. *Colorectal Dis* 2017; **19**: 907–911. 28444968 10.1111/codi.13695

[11] Rasul S, Hartenbach S, Rebhan K *et al.* 18FDOPA PET/CeCT in diagnosis and staging of primary medullary thyroid carcinoma prior to surgery. *Eur J Nucl Med Mol Imaging* 2018; **45**: 2159–2169. 29766245 10.1007/s00259-018-4045-9 PMC6182401

[12] National Institute for Health Research (NIHR). *Interactive costing tool (iCT). Investigation and intervention tariff 2020/21 – RT009, RT010*. Version 1.2 – 14th April 2020. https://www.leedsth.nhs.uk/assets/71432c14fa/NIHR-2020-Investigation-and-Intervention-Tariff-1-v2.2-1.pdf (cited August 2023).

